# Gait parameters when walking with or without rollator on different surface characteristics: a pilot study among healthy individuals

**DOI:** 10.1186/s13104-022-06196-9

**Published:** 2022-09-24

**Authors:** Eva Ekvall Hansson, Yara Akar, Tingting Liu, Cong Wang, Agneta Malmgren Fänge

**Affiliations:** 1 Department of Health Sciences, Human Movement: Health and Rehabilitation, Lund University, Lund, Sweden; 2 Department of Health Sciences, Lund University, Lund, Sweden; 3 Department of Health Sciences, Applied Gerontology, Lund University, Lund, Sweden

**Keywords:** Gait, Rollator, Step length, Walking speed, Sideway deviation, Environmental condition

## Abstract

**Objectives:**

Gait parameters can measure risks of falling and mortality and identify early stages of frailty. The use of walking aid changes gait parameters. The aim of this study was to describe differences in gait parameters among healthy adults when walking on different surfaces and under different conditions, with and without a rollator.

**Results:**

Ten healthy participants walked first without and then with a rollator upslope, downslope and on flat surface, on bitumen and gravel respectively. Step length, walking speed and sideway deviation was measured using an inertial measurement unit. Walking up a slope using a rollator generated the longest step length and walking down a slope using a rollator the shortest. Fastest walking speed was used when walking up a slope with rollator and slowest when walking down a slope with rollator. Sideway deviation was highest when walking down a slope and lowest when walking on gravel, both without rollator. Highest walk ratio was found when walk up a slope without rollator and lowest when walking down a slope with rollator. Data from this study provides valuable knowledge regarding gait parameters among healthy individuals, useful for future clinical research relevant for rehabilitation and public health.

## Introduction

Mobility is a prerequisite for the vast majority of everyday activities, and being able to move around outdoors contributes to societal participation [1, 2], and is known to reduce depression and social stigma [3]. Increasing age is associated with physical activity limitations due to illness, injury or general age-related health decline [4]. Walking is an easily accessible way of performing physical activity. To be able to walk as fast as required to gain health benefits, walking outdoors is usually necessary. Walking outdoors is however a complex task that places demand on e.g., muscle strength, balance, and cognition. Difficulties to walk may be caused by possible transportation barriers, walking distance, having to manage walking in traffic with time limits (crossing the street on signals), maneuvering among people, bicycles or other objects, difficult terrains, and the rapid changes of the environment [5]. Different outdoor surfaces can be perceived as challenging depending on the complexity of the surface [6]. Therefore, to measure gait parameters included in walking outdoors and in different terrains can be of interest. Gait parameters are often used to measure risks of falling, and mortality, and identify early stages of frailty [7]. Thus, it must be understood how gait can be associated with these clinical features, to come up with preventive measures.

Among persons with reduced ability to walk, walking aids are often used to enable activity and participation [8]. Even if a walking aid helps in reducing dependency and increases functionality in the environment [9], it also changes gait. For example, the use of a rollator increases in particular gait velocity and step length among older persons compared to when walking without a rollator [10]. Older persons who use rollator, especially women, are highly overrepresented in single accidents in traffic [11].Thus, gait parameters when using a walking aid is also of interest, and for a clinician to successfully identify what a dysfunctional gait is, first, one must be able to readily define what a normal gait is both with and without a walking aid. Thus, after defining a normal gait with its general population-based parameters, the clinician knows what to expect and what to look for [7]. Knowledge concerning gait parameters among healthy persons can also be useful when planning rehabilitation focusing on regaining activity and to enable participation in the society.

Thus, the aim of this study was to describe differences in gait parameters among healthy adults when walking on different surfaces and under different conditions, with and without a rollator.

## Main text

### Materials and methods

The study was an experimental, cross-sectional pilot study. Participants was recruited among students and staff at Lund University, using the following inclusion criteria: 18 years or older; consider themselves healthy; and without known health conditions that could impact their mobility. No other inclusion or exclusion criteria were used. 10 participants were included; seven women and three men, aged 23–62 years (Median 35 years).

#### Measures

Gait was measured with a nine-axis Inertial Measurement Unit (IMU), worn on the right thigh. The IMU includes an accelerometer, magnetometer, and gyroscope that can detect and measure movement patterns when changing body positions in the frontal, sagittal and mediolateral planes and analyze gait. The IMU is comprised by an STM32 microprocessor coupled to an LSM6DSM accelerometer/gyroscope combination, sampling apparent acceleration and rotational velocity at 50 Hz. The IMU also comprises a GPS and a clock. IMU:s is a practical and portable way of collecting this type of data [12] and can be used for analyzing a range of components of movements [13]. The IMU used in this project was custom-built, with project-specific measurements such as sideways deviation, but made use of the proprietary Snubblometer library from Infonomy AB for step detection and gait classification (www.infonomy.com). The IMU has shown good validity and reliability for measuring postural sway [14], for detecting a near-fall [15] and good ability to identify future fallers [16]. The hardware and the algorithms for determining step length, step time and walking speed are identical to those used in earlier validation experiments, where excellent validity and reliability has been shown [17, 18], although the IMU used in this study has had some additional features implemented for this measurement.

Gait was measured using four parameters: step length, walking speed, sideway deviation and walk ratio. The direction of measured progression was forward. Step length is defined as step in cm, which is a half gait cycle (right heel strike to left heel strike). Step length is estimated from the angle of the thigh in each completed stride-cycle. In this approximation, a 10 degrees angle in the knee when the heel strikes the floor is assumed [19]. Walking speed is calculated by stride length divided in stride duration and is measured in m/s. The project in question was considered exploratory, and therefore the sensor’s frame of reference was used to calculate sideways deviation as a catch-all for both translational movement and rotation in the mediolateral direction. These two movement types are closely related, as a significant sideways step always corresponds to an outward or inward rotation of the thigh. In sideway deviation, the discretized (at 50 Hz) time-integral of the absolute value of the apparent acceleration along the Z-axis (aligned with the mediolateral axis of the body) was calculated for each step and divided by the total step duration to calculate an arithmetic mean of the absolute value of the Z-acceleration during the step. This measure functions as a flag for steps comprising a high degree of sideways movement, and because it is calculated by the absolute value, it will register sideways movement regardless of whether the movement is towards the right, towards the left, or whether the acceleration during a particular step is monodirectional or bidirectional (positive acceleration followed by negative acceleration, or vice versa, which would cancel each other out if the integral was not of the absolute value). The measure was described as “sideways deviation” for simplicity, as it is analogous to a deviation from the centre line of the gait movement, but as the accelerometer measures apparent acceleration rather than true acceleration, a high value can also be caused by a large shift in sideways inclination, without much translational movement. Walk ratio is defined as step length divided in cadence [20]. The measure of walk ratio was added post-hoc and is therefore not normalized for height.

The gait parameters were measured during walk under two different conditions: with and without the use of a four wheeled rollator. In addition, gait was measured for each condition on different surface characteristics: up-slope, down-slope, bitumen, and gravel paths. All up-slope and down-slope sections were paved with bitumen, while all gravel paths were flat.

#### Procedure

The outdoor walk consisted of a lap that partly went in parallel to a bicycle route, and several streets were crossed, including tramrails and traffic lights. Two observants noted the exact time when the environmental condition changed, for example the beginning and end of a gravel part of the lap. Each lap was walked twice, first without rollator, and secondly with rollator. Before starting the lap with rollator, the participants walked for a few minutes to get accustomed to it. They were also informed about the properties of the rollator, and it was adjusted to each participants height. The lap was in total 2.5 km long, with a gravel part of approx. 600 m in the first half the lap. The slope was 100 m long, with an inclination of 1:40. Each lap took 30–45 min to finish. The participants had a convenience break between the two laps, approx. 5–10 min.

Data were collected during February–March 2021. Thus, the weather conditions varied from dry to wet conditions, with a temperature between 0–15 degrees Celsius.

#### Data analysis and statistics

IBM SPSS version 25 was used for statistical analyses. The data generated by the IMU were recorded each second and analyzed according to the different environmental characteristics in the outdoor walk, with and without rollator. Since not all variables were normally distributed and sample size was small, both mean (M) and standard deviation (SD) as well as median (Md) and min–max values were used. Since repeated measures was applied, an analysis of variance was performed, using repeated measures two-way ANOVA. A p ≤ 0.05 was used for determining statistical significance [21].

Calculating with minimal clinical statistical difference of 0.1 m/sec in walking speed [22], with a standard difference of 0.15 m/sec [23] and statistical significance of 0.05, a sample size in a full-scale study was set to 70 participants. In this pilot-study, sample size was estimated to about 15% of the full-scale study, that is 10 participants.

### Results

**Fig. 1:**
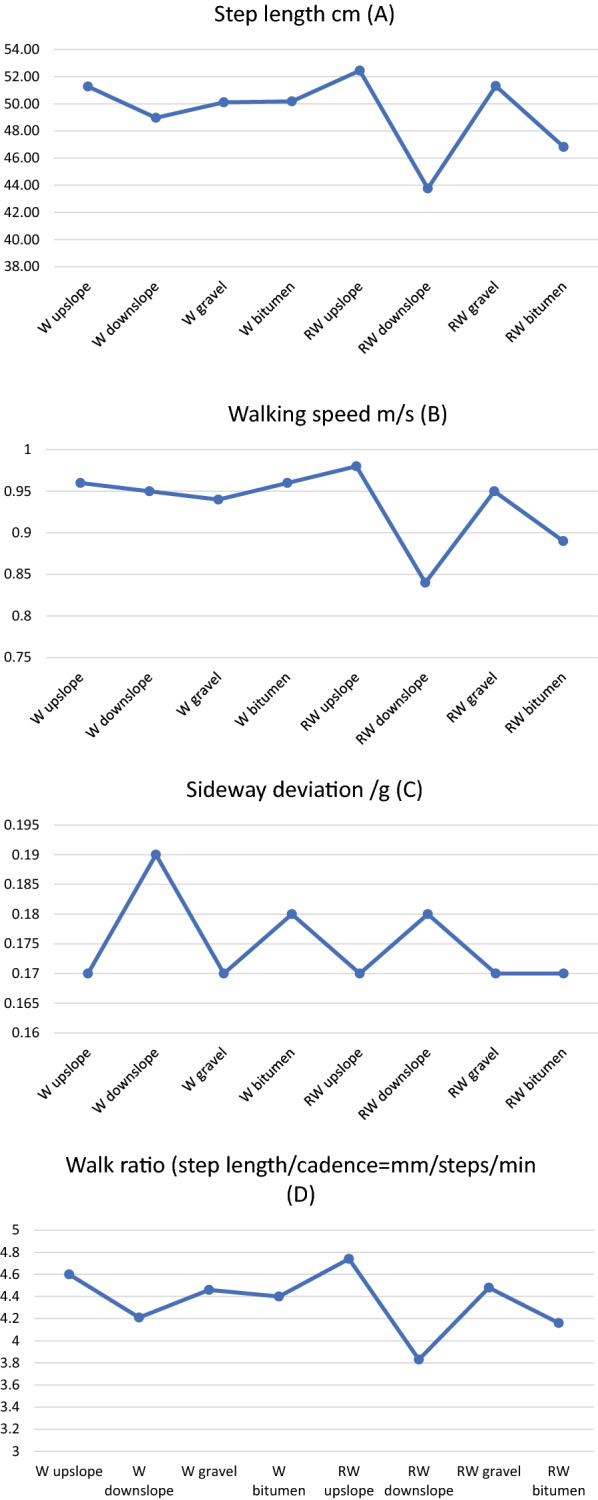
A-D. The figure displays step length in cm (**A**), walking speed in m/s (**B**), sideway deviation in g (**C**) and walk ratio in mm/steps/min when walking with and without rollator under different conditions

Both the longest and shortest steps were taken while walking with a rollator, with the longest step length used when walking upslope (M = 52.44 cm, Md = 54.87 cm) and shortest step length was used when walking downslope (M = 43.77 cm, Md = 41.04). The highest and lowest walking speed was also identified when the rollator was used. The highest speed was measured when walking upslope with rollator (M = 0.98 m/s, Md = 1.07 m/s) and the lowest speed when walking downslope with rollator (M = 0.84 m/s, Md = 0.75 m/s). Highest sideway deviation was found when walking downslope, without rollator (M = 0.19 g, Md = 0.18 g) and lowest sideway deviation was found when walking on gravel without rollator (M = 0.17 g, Md = 0.16 g). Highest walk ratio was found when walking up a slope without a rollator M = 4.60, Md = 4.59) and lowest when walking down a slope with a rollator (M = 3.83, Md = 3.62). Mean and median values and SD and min–max values for the different variables when walking in the different terrains, with and without rollator are displayed in Table 1. Mean values are also shown in Fig. 1A–C.

**Table 1:** Descriptive statistics of gait parameters when walking on different surfaces and during different conditions N = 10

Walking conditions	Step length/cm		Walking speed m/s	
	Mean (SD)	Median (min–max)^*^	Mean (SD)	Median (min–max)
Walk upslope	51.27 (7.88)	51.20 (41.13–60.73)	0.96 (0.15)	0.97 (0.75–1.17)
Walk downslope	48.97 (8.36)	50.83 (38.00–59.22)	0.95 (0.19)	0.95 (0.70–1.19)
Walk on gravel	50.11 (7.17)	48.42 (38.60–60.36)	0.94 (0.15)	0.94 (0.70–1.11)
Walk on bitumen	50.18 (8.04)	51.01 (37.67–62.22)	0.96 (0.16)	0.99 (0.69–1.17)
Rollator walk upslope	52.44 (9.47)	54.87 (40.25–62.84)	0.98 (0.22)	1.07 (0.68–1.25)
Rollator walk downslope	43.77 (10.01)	41.04 (33.22–61.10)	0.84 (0.25)	0.75 (0.59–1.29)
Rollator walk on gravel	51.31 (8.33)	51.15 (36.43–60.19)	0.95 (0.21)	0.92 (0.67–1.31)
Rollator walk on bitumen	46.82 (8.59)	45.36 (32.51–57.42)	0.89 (0.22)	0.85 (0.61–1.19)

	Sideway deviation/g		Walk ratio mm/steps/min	
Walk upslope	0.17 (0.03)	0.17 (0.12–0.23)	4.60 (0.76)	4.59 (0.37–5.44)
Walk downslope	0.19 (0.03)	0.18 (0.14–0.25)	4.21 (6.53)	4.34 (3.43–5.29)
Walk on gravel	0.17 (0.03)	0.16 (0.12–0.22)	4.46 (6.64)	4.24 (3.47–5.56)
Walk on bitumen	0.18 (0.03)	0.17 (0.12–0.22)	4.40 (7.14)	4.26 (3.42–5.50)
Rollator walk upslope	0.17 (0.03)	0.17 (0.12–0.23)	4.74 (88.01)	4.90 (3.45–6.26)
Rollator walk downslope	0.18 (0.04)	0.18 (0.13–0.25)	3.83 (7.42)	3.62 82.98–4.92
Rollator walk on gravel	0.17 (0.03)	0.18 (0.13–0.22)	4.48 (6.99)	4.64 (3.16–5.31)
Rollator walk on bitumen	0.17 (0.04)	0.17 (0.13–0.25)	4.16 (6.48)	4.28 (2.87–5.10)

^*^Since not all variables were normally distributed and the sample size is small, both mean and median values are shown

For step length, the analysis of variance during the different conditions showed that walking downslope with a rollator differed significantly from five of the other conditions (p ≤ 0.00–0.01) and walking without a rollator on gravel did not differ from any of the other conditions (Table 2).

**Table 2:** Mean differences, standard deviaton (SD) and p-values for the differences in step length, walking speed, sideway deviation and walk ratio between the different walking conditions and surfaces, N = 10

	Walk upslope^1^	Walk downslope^1^	Walk on gravel^1^	Walk on bitumen^1^	Rollator walk upslope	Rollator walk downslope	Rollator walk on gravel	Rollator walk on bitumen
Step length in cm								
Walk upslope		2.31 (5.74) 0.24	1.16 (3.56) 0.33	1.09 (3.79) 0.39	1.17 (9.00) 0.69	7.50 (6.09) **0.01**	0.95 (6.85) 0.67	4.45 (0.09) 0.68
Walk downslope	2.31 (5.74) 0.24		1.14 (3.58) 0.34	1.21 (3.79) 0.34	3.47 (8.74) 0.24	5.20 (5.44) **0.01**	1.35 (4.24) 0.34	2.15 (5.28) 0.23
Walk on gravel	1.16 (3.56) 0.33	1.14 (3.58) 0.34		0.08 (3.03) 0.94	1.14 (3.58) 0.39	6.33 (6.41) 0.12	0.21 (5.49) 0.91	3.29 (6.26) 0.13
Walk on bitumen	1.09 (3.79) 0.39	1.21 (3.79) 0.34	0.08 (3.03) 0.94		2.26 (9.18) 0.45	6.41 (6.00) **0.01**	0.13 (5.13) 0.94	3.36 (5.63) 0.09
Rollator walk upslope	1.17 (9.00) 0.69	3.47 (8.74) 0.24	2.34 (8.14) 0.39	2.26 (9.18) 0.45		8.67 (7.47) **0.01**	2.13 (6.26) 0.31	5.62 (6.60) 0.25
Rollator walk downslope	7.50 (6.09) **0.01**	5.20 (5.44) **0.01**	6.33 (6.41) 0.12	6.41 (6.00) **0.01**	8.67 (7.47) **0.01**		6.54 (5.22) ** ≤ 0.00**	3.05 (4.40) 0.06
Rollator walk on gravel	0.95 (6.85) 0.67	1.35 (4.24) 0.34	0.21 (5.49) 0.91	0.13 (5.13) 0.94	2.13 (6.26) 0.31	6.54 (5.22) ** ≤ 0.00**		3.49 (2.08) ** ≤ 0.00**
Rollator walk on bitumen	4.45 (0.09) 0.68	2.15 (5.28) 0.23	3.29 (6.26) 0.13	3.36 (5.63) 0.09	5.62 (6.60) 0.25	3.05 (4.40) 0.06	3.49 (2.08) ** ≤ 0.00**	
Walking speed in m/s								
Walk upslope		0.01 (0.09) 1.00	0.02 (0.06) 1.00	0.01 (0.06) 1.00	0.02 (0.17) 1.00	0.12 (0.15) 0.91	0.01 (0.15) 1.00	0.07 (0.15) 1.00
Walk downslope	0.01 (0.09) 1.00		0.01 (0.08) 1.00	0.01 (0.07) 1.00	0.03 (0.18) 1.00	0.11 (0.13) 0.62	0.00 (0.18) 1.00	0.06 (0.13) 1.00
Walk on gravel	0.02 (0.06) 1.00	0.02 (0.08) 1.00		0.02 (0.06) 1.00	0.04 (0.17) 1.00	0.04 (0.17) 1.00	0.01 (0.15) 1.00	0.05 (0.16) 1.00
Walk on bitumen	0.01 (0.06) 1.00	0.01 (0.07) 1.00	0.02 (0.06) 1.00		0.02 (0.17) 1.00	0.12 (0.15) 0.91	0.01 (0.13) 1.00	0.07 (0.14) 1.00
Rollator walk upslope	0.02 (0.17) 1.00	0.03 (0.18) 1.00	0.04 (0.17) 1.00	0.02 (0.17) 1.00		0.14 (0.14) 0.34	0.03 (0.12) 1.00	0.09 (0.11) 0.74
Rollator walk downslope	0.12 (0.15) 0.91	0.11 (0.13) 0.62	0.10 (0.17) 1.00	0.12 (0.15) 0.91	0.14 (0.14) 0.34		0.11 (0.11) 0.37	0.05 (0.09) 1.00
Rollator walk on gravel	0.01 (0.15) 1.00	0.00 (0.18) 1.00	0.01 (0.15) 1.00	0.01 (0.13) 1.00	0.03 (0.12) 1.00	0.11 (0.11) 0.37		0.06 (0.04) **0.03**
Rollator walk on bitumen	0.07 (0.15) 1.00	0.06 (0.13) 1.00	0.05 (0.16) 1.00	0.07 (0.14) 1.00	0.09 (0.11) 0.74	0.05 (0.09) 1.00	0.06 (0.04) **0.03**	
Sideway deviation/g*								
Walk upslope		0.01 (0.02) 0.43	0.01 (0.01) 0.23	0.00 (0.01) 0.54	0.00 (0.02) 0.57	0.01 (0.02) 0.24	0.00 (0.02) 0.61	0.00 (0.03) 0.76
Walk downslope	0.01 (0.02) 0.43		0.02 (0.01) ** ≤ 0.00**	0.00 (0.01) ** ≤ 0.00**	0.02 (0.03) 0.62	0.01 (0.02) 0.49	0.01 (0.02) **0.02**	0.01 (0.02) 0.11
Walk on gravel	0.01 (0.01) 0.23	0.02 (0.01) ** ≤ 0.00**		0.01 (0.01) **0.01**	0.00 (0.02) 0.65	0.02 (0.03) 0.57	0.00 (0.02) 0.48	0.01 (0.02) 0.25
Walk on bitumen	0.00 (0.01) 0.54	0.00 (0.01) ** ≤ 0.00**	0.01 (0.01) ** ≤ 0.00**		0.01 (0.01) 0.40	0.01 (0.02) 0.25	0.01 (0.01) 0.33	0.00 (0.00) 0.95
Rollator walk upslope	0.00 (0.02) 0.57	0.02 (0.03) 0.62	0.00 (0.02) 0.65	0.01 (0.01) 0.40		0.13 (0.02) **0.05**	0.00 (0.01) 0.75	0.01 (0.02) 0.30
Rollator walk downslope	0.01 (0.02) 0.24	0.01 (0.02) 0.49	0.02 (0.03) 0.57	0.01 (0.02) 0.25	0.13 (0.02) **0.05**		0.01 (0.14) **0.02**	0.01 (0.01) 0.15
Rollator walk on gravel	0.00 (0.02) 0.61	0.01 (0.02) **0.02**	0.00 (0.02) 0.48	0.01 (0.01) 0.33	0.00 (0.01) 0.75	0.01 (0.14) **0.02**		0.00 (0.01) 0.23
Rollator walk on bitumen	0.00 (0.03) 0.76	0.01 (0.02) 0.11	0.01 (0.02) 0.25	0.00 (0.00) 0.95	0.01 (0.02) 0.30	0.01 (0.01) 0.15	0.00 (0.01) 0.23	
Walk ratio mm/steps/min								
Walk upslope		3.85 (6.17) 0.08	1.39 (4.05) 0.31	1.96 (4.41) 0.19	1.40 (9.36) 0.65	3.84 (6.17) ≤ **0.00**	1.19 (6.79) 0.59	4.41 (6.12) **0.05**
Walk downslope	3.85 (6.17) 0.08		2.46 (3.19) **0.04**	1.89 (3.95) 0.16	5.25 (7.87) 0.06	3.82 (3.79) **0.01**	2.65 (3.31) **0.03**	1.89 (3.95) 0.59
Walk on gravel	1.39 (4.05) 0.37	2.46 (3.19) **0.04**		0.57 (2.93) 0.55	2.79 (7.46) 0.27	6.29 (3.61) ≤ **0.00**	0.88 (3.60) 0.87	3.02 (3.63) **0.03**
Walk on bitumen	1.96 (4.41) 0.19	1.89 (3.95) 0.16	0.57 (2.93) 0.55		3.36 (8.87) 0.26	5.71 (4.17) **0.02**	0.57 (2.93) 0.61	2.49 (4.25) 0.09
Rollator walk upslope	1.40 (9.36) 0.65	5.25 (7.87) 0.06	2.79 (7.46) 0.27	3.36 (8.87) 0.26		9.07 (7.20) ** ≤ 0.00**	2.59 (5.81) 0.19	5.85 (6.73) **0.02**
Rollator walk downslope	3.84 (6.17) ≤ **0.00**	3.82 (3.79) **0.01**	6.29 (3.61) ≤ **0.00**	5.71 (4.17) **0.02**	9.07 (7.20) **0.03**		6.48 (4.59) **0.01**	3.22 (3.51) **0.02**
Rollator walk on gravel	1.19 (6.79) 0.59	2.65 (3.31) **0.03**	0.88 (3.60) 0.87	0.57 (2.93) 0.61	2.59 (5.81) 0.19	6.48 (4.59) ≤ **0.00**		3.26 (2.36) ≤ **0.00**
Rollator walk on bitumen	4.41 (6.12) **0.05**	1.89 (3.95) 0.59	3.02 (3.63) **0.03**	2.49 (4.25) 0.09	5.85 (6.73) **0.02**	3.22 (3.51) **0.02**	3.26 (2.36) ≤ **0.00**	

P-value calculated by repeated measures two-way ANOVA. Statistically significant p-values are displayed in bold

^*^g gravitational acceleration

^1^Mean diff (SD)

For walking speed, the analysis of variance showed that the walking conditions did not differ, except between walking with a rollator on gravel and with a rollator on bitumen (p = 0.03) (Table 2).

For sideway deviation, the analysis showed that walking downslope without a rollator differed significantly from four of the other conditions (p ≤ 0.00–0.04) and walking upslope without a rollator and on bitumen with a rollator did not differ from any of the other conditions (Table 2).

For walk ratio, the analysis showed that walking down a slope with a rollator differed significantly from all of the other conditions (p = 0.00–0.03) (Table 2).

### Discussion

This pilot study showed that step length, walking speed, sideway deviation and walk ratio can differ when walking in different terrains and with and without walking. Walking up a slop using a rollator demanded longest step length and walking down a slope using a rollator the shortest. Fastest walking speed was used when walking up a slope using a rollator and slowest when walking down a slope using a rollator while sideway deviation was highest when walking down a slope and lowest when walking on gravel, both without rollator. Walk ratio was highest when walking up a slope without a rollator and lowest when walking down a slope with a rollator. Walking down a slope with rollator differed the most between the other conditions in step length and in walk ratio, walking downslope without rollator differed the most from the other conditions in sideway deviation. There were few differences between the conditions in walking speed.

Other research has shown an increase in step length among older persons when walking with a four-wheeled rollator on plain ground [24]. We therefore expected to find more differences between walking with at rollator on bitumen than we did, which might depend on our sample of healthy, younger persons. Using wearable sensors to measure gait with walking aid can reduce the accuracy of the measure, which also could explain the small differences [25]. However, step length is related to fear of falling among older adults [26] and fear of falling and falls are also related [27]. Thus, including measures of step length when planning rehabilitation programs for older adults, with the aim to increase gait performance and reduce risk of falls might be relevant. Sideway deviation can be related to the ability to vary gait during different conditions, which in turn has a relation to falls [16]. Thus, our findings on highest sideway deviation when walking downslope can be an indication on walking downslope being especially challenging for balance.

Walking speed has a relationship to mortality and can be used as a marker of physical performance and therefore be clinically useful [28, 29]. Our results showed very few differences in walking speed between the different conditions and further studies, measuring gait parameters among older persons during different conditions is called upon.

Step length, speed and sideway deviation during walking can differ depending on rollator use, walking surface and environmental characteristics, at least among healthy individuals. Walking downslope with a rollator generated most differences in step length compared to the other conditions and sideway deviation was largest when walking downslope without a rollator. Walking speed however showed very few differences between the conditions. Also, walking upslope with a rollator generated the longest steps (52.44 cm) and walking downslope with a rollator the shortest (43.77 cm). That means that pushing the rollator upslope demands more energy and therefore both step length and walk ratio possibly is increased, and vice versa going down the slope: to slow down speed downslope also demands more energy and step length as well as walk ratio therefore has to be reduced. This implies that walking up and down slopes can be challenging for balance and therefore important to include in rehabilitation programs directed towards improving balance. The type of gait parameters used in this study can be easily measured using wearable devices, such as IMU:s.

Our findings provide valuable knowledge regarding gait parameters among healthy individuals, useful for future clinical research relevant for rehabilitation planning and evaluation. The findings are also valuable for the design of pedestrian routes in urban areas to support physical activity and participation in the population.

### Limitations

The study has several limitations. To be able to compare walking in the different conditions people who considered themselves healthy were enrolled in this study. This approach has been used before, since it is difficult, or even unethical, to ask persons who are dependent on a walking aid, to walk without it [30]. Gait velocity and stride length have however been shown to differ between first time users of a different types of walking aid, such as rollators, crutches and three-wheeled walkers, and frequent users [10]. Research measuring gait parameters when walking in different terrains and under different conditions, including people with disabilities is needed.

Walk ratio was included as a post-hoc analysis and is therefore not normalized for height, since height was not measured on the participants. This must be taken into account when interpreting our results on walk ratio.

Even if sufficient for the study design applied, the sample size was small [31, 32], which of course limits the ability to draw conclusions. We aimed to include a larger number of participants, however, due to the COVID-19 restrictions in Sweden, this was not possible. Instead, we extended the walk so that the loop included a large variety of different surfaces and environmental conditions. Thus, we managed to collect a large amount of data from each participant which increased the ability to draw some preliminary conclusions [33]. Another limitation is that we used only one IMU to collect data for each participant. Using additional IMU:s is for example reliable for measuring trunk range of motion [34] and to detect anticipatory postural adjustments [35]. Instrumental footwear has also shown to be feasible for ambulatory gait analysis [25]. However, we used the same lap in all walks, which increases reliability of the data collected.

In future research, the use of several IMU’s can extend the type of data that can be collected in this type of experimental studies and thereby expand knowledge on which type of movements that are necessary for a person to manage to be able to walk outdoors. In this aspect, research regarding gait parameters among older people and people with disabilities is also important.

## Data Availability

Data will be available upon reasonable request to the corresponding author.

## Abbreviations

IMU: Inertial measurement unit
M: Mean
SD: Standard deviation
Md: Median
